# Glucose Tolerance and Weight Loss in Obese Women with Obstructive Sleep Apnea

**DOI:** 10.1371/journal.pone.0061382

**Published:** 2013-04-17

**Authors:** Luisa Gilardini, Carolina Lombardi, Gabriella Redaelli, Luciana Vallone, Andrea Faini, Paola Mattaliano, Gianfranco Parati, Cecilia Invitti

**Affiliations:** 1 Department of Medical Sciences and Rehabilitation, Istituto Auxologico Italiano, Milan, Italy; 2 Sleep Disorders Center, Department of Cardiology, Istituto Auxologico Italiano, Milan, Italy; 3 Department of Clinical Medicine and Prevention, University of Milano-Bicocca, Milan, Italy; Azienda Policlinico S. Orsola-Malpighi, Italy

## Abstract

**Background:**

The association of obstructive sleep apnea (OSA) with glucose intolerance and the beneficial effect of lifestyle intervention have been poorly investigated in women particularly before menopausal status. The study explored 1) whether OSA is associated with impaired glucose homeostasis in obese non diabetic premenopausal and menopausal women and 2) the effects of a 3- month lifestyle intervention on glucose homeostasis in OSA women.

**Design and Methods:**

We consecutively recruited 98 obese women (39 premenopausal) from those referred for a weight loss intervention. Ambulatory nocturnal polysomnography, body composition, oral glucose tolerance test, insulin sensitivity and β cell function were assessed before and after intervention.

**Results:**

41% of premenopausal and 64% of menopausal women had OSA which was associated with worse glucose homeostasis before menopausal status. Mean and minimal nocturnal oxygen saturation (SaO_2_) was associated with neck/height ratio (NHR), independently of total and central obesity. Mean and minimal nocturnal SaO_2_ and NHR were correlated with insulin sensitivity and fasting glucose. In multivariate analyses, nocturnal mean SaO_2_ was negatively and independently correlated with fasting glucose (p<0.0001) and NHR with insulin sensitivity (p<0.0001). In OSA women, the intervention induced a 5% weight reduction and a significant increase in minimal nocturnal SaO_2_, insulin sensitivity and β cell function. Changes in fasting glucose and insulin sensitivity were associated with those in minimal nocturnal SaO_2_ (p<0.05) and not with weight loss.

**Conclusions:**

In obese women, glucose homeostasis worsens due to nocturnal hypoxia and increased neck circumference through mechanisms partially independent of obesity. OSA is more clearly associated with glucose intolerance in premenopausal than in menopausal women. In OSA women, the improvement of nocturnal hypoxia induced by lifestyle modifications is associated with that of glucose homeostasis.

## Introduction

Obstructive sleep apnea (OSA) is a serious widespread disease in which upper airways undergo repeated occlusions and arterial oxygen saturation (SaO_2_) repeatedly falls during sleep. OSA defined by a number of significant apnea/hypopnea index episodes ≥5 per hour, is estimated to occur in approximately 24% of men and 9% of women in the general population [1]. OSA is a risk factor for cardiovascular disease and type 2 diabetes [2], [3]. All these diseases are predicted by weight gain [4], but whether the association of OSA with diabetes is due to the coexisting obesity, this remains to be determined [3].

In 2008 the International Diabetes Federation recommended to raise awareness of possible OSA in patients with type 2 diabetes as well as to look for metabolic disorders in patients with OSA [5]. This recommendation was built on the high prevalence of type 2 diabetes in patients with OSA and on the direct relation between glucose tolerance and OSA severity ([3] for review). Several cross-sectional and longitudinal studies, however, were unable to conclude unequivocally that OSA is an independent risk factor for diabetes [3], [6], [7]. Furthermore, in type 2 diabetic patients with OSA who underwent lifestyle intervention, changes in glucose control were determined by changes in weight rather than in AHI [8] and the beneficial effects of CPAP treatment on glucose tolerance are controversial [3].

Similarly to what observed for type 2 diabetes, the prevalence of impaired fasting glucose (IFG) and of impaired glucose tolerance (IGT) increase with the severity of OSA [9]–[12]. These prediabetic states are characterized by a decrease in insulin sensitivity which has been independently associated with the presence and severity of OSA in several population and clinic-based cross-sectional studies [3], [12], [13]. Other studies however, reported that in OSA patients insulin resistance and metabolic syndrome are determined by obesity rather than by the characteristic sleep breathing disorder [14]–[17]. Besides obesity, other factors like age and gender may confound the relation of OSA with glucose homeostasis. For example, most studies have been conducted in men because they suffer more frequently of sleep-disordered breathing [1]. However, men and women are alike for the role of abdominal obesity on metabolic consequences as well as for alterations in insulin sensitivity and in glucose control [18], [19].

Strategies to modify lifestyle habits are encouraged for the treatment of sleep-disordered breathing and very low energy diets or bariatric surgery have been shown to reduce AHI by 40 and 70% respectively [20], [21]. To date studies evaluating the effects of a moderate weight loss, easy to implement in the clinical practice, in ameliorating cardiovascular risk factors in OSA are few. Furthermore, most of these studies were carried out in men and showed doubtful benefits in women ([22] for review).

Therefore given that 1) uncertainty exists concerning the independent association of OSA with abnormal glucose tolerance particularly in women [11], [23]; 2) the relation between changes in glucose tolerance and in OSA severity after weight loss has been poorly investigated, we analyzed whether OSA is associated with impaired glucose homeostasis in premenopausal (PM) and menopausal (M) non diabetic obese women. The relationship between changes in nocturnal polysomnographic data and glucose homeostasis after a 3-month lifestyle intervention were also examined.

## Patients and Methods

### Ethic Statement

The study was approved by the Ethics Committee of Istituto Auxologico Italiano, and written informed consent was obtained from all subjects after a full explanation of the study methods and purposes.

The study sample consisted in 98 Caucasian non diabetic obese women consecutively recruited during the second half of 2010 from those referred to the Istituto Auxologico Italiano for a weight-loss lifestyle intervention. We enrolled non diabetic women because the relationships of OSA with alterations in glucose homeostasis are less likely to be blurred by confounding factors as in the overt diabetes.

In all women, information on smoking habits, use of medications and family history of diabetes was collected. Menopausal status was defined by self-reported amenorrhea for at least 12 months.

Excessive daytime somnolence was assessed before and after the intervention using the Epworth Sleepiness Scale [24], with a score threshold of more than 10 been taken as suggestive for excessive daytime somnolence.

Before and 3 months after lifestyle intervention, anthropometric measures, blood pressure (BP) and heart rate were measured and body composition assessed by bioelectric impedance assay (BIA 101-RJL Systems Akern srl, Firenze, Italy). The neck circumference was measured just below the laryngeal prominence and waist circumference at the level of the umbilicus. The oral glucose tolerance test (OGTT) was performed with the measurement of glucose and insulin and a fasting blood sample taken for C-reactive protein (CRP) measurements. Insulin sensitivity was estimated using the OGTT-derived insulin sensitivity index (ISI) which is a marker of whole body (muscle plus liver) insulin sensitivity and strongly correlates with the results obtained with the euglycemic insulin clamp [25]. ISI was computed according to the formula: 10000/√ ((fasting glucose×fasting insulin)×((glucose0*15+ glucose30*30+glucose60*30+glucose90*30+glucose120*15)/120)×((insulin0*15+insulin30*30+insulin60*30+insulin90*30+insulin120*15)/120)). Beta cell function was determined by the insulinogenic index (ΔI_30_/ΔG_30_) expressed as the ratio of the incremental (0–30 min) insulin and glucose response to OGTT [26]. Impaired fasting glucose (IFG) and impaired glucose tolerance (IGT) were defined using the American Diabetes Association criteria. Clinic BP was measured in a sitting position, three times every 5 min with a standard mercury sphygmomanometer and a cuff size optimized for arm circumference. Phase I and V (disappearance) Korotkoff sounds were used to identify systolic and diastolic BP. Obese women were classified as hypertensive if they had clinic BP>140/90 or were on antihypertensive therapy.

Lifestyle intervention lasted 3 months and consisted in weekly visits to our center for nutritional education, advice reinforcement on exercise activity and peer group psychological support. A self-monitor diary including food consumption, daily physical activity and emotional reactions was used as a tool for education and reinforcement. Daily caloric requirements were calculated by using the Harris-Benedict equation and an individual activity factor. Diet instructions based on a 750-kcal/d deficit from estimated caloric requirement (1200–2000 kcal/day) were given by a dietician to each subject. Diet included 17–22% of total energy intake as protein, 23–25% as fat and 55–58% as carbohydrate. A physical activity program was prescribed consisting in 210 minutes per week (70% of moderate-intensity aerobic physical activity and 30% of muscle-strengthening activities).

### Biochemical Measurements

Circulating levels of glucose were measured using an automated analyzer (Roche Diagnostic, Manheim, Germany). Insulin was measured by Electrochemiluminescence ImmunoAssay (Roche Diagnostic, Monza, Italy) with a detection limit of 0.2 mU/L and intra- and inter-assay CV of 0.9% and 3.4%, respectively. CRP concentrations were measured by immunoturbidimetric assay (Roche Diagnostics, Monza, Italy) with a detection limit of 0.3 mg/L and intra- and inter-assay CV of 1.1% and 3.3%.

### Ambulatory Nocturnal Polysomnography (Cardio-respiratory Monitoring)

All obese women underwent ambulatory nocturnal polysomnography before and after 3 months of lifestyle intervention. For at least 8 hours, including at least 4 hours of sleep, the following parameters were recorded: ECG, nasal airflow, thoracic and abdominal effort, oxygen saturation by pulse oximetry, snoring and body position. An OSA event was defined as a period of 10 or more seconds of complete nasal airflow cessation during sleep; hypopnea as a period equal to or longer than 10 seconds of discernible reduction in airflow, accompanied by a SaO2 reduction ≥4%. According to International Guidelines OSA is considered mild when AHI is between 5 and 15, moderate with an AHI between 15 and 30 and severe when AHI is above 30 events per hour [27].

### Statistical Analyses

Variables that were not normally distributed were log transformed for the analysis.

To minimize the possible positive effects of height on cardio-metabolic morbidity, in the analyses we used the waist-to-height ratio (WHR), as well as the neck-to-height ratio (NHR). Two sample t-tests and ANCOVA were used to examine the differences between groups. Frequencies were compared using a χ^2^ test. Pearson correlation analyses were used to evaluate bivariate relationships. Partial Correlations procedure was used to describe the linear relationship between two variables while controlling for the effects of one or more additional variables.

Multivariate regression analysis was performed using variables statistically significant at the 5% level in univariate analysis. Changes induced by lifestyle intervention were calculated as the ratio between 3-month value less baseline value and baseline value. Test t for one sample was used to analyze changes from baseline. A probability value <0.05 was considered significant. Data are given as the means ± SD unless otherwise stated. All analyses were performed using SPSS version 19.0 (SPSS, Chicago, IL, US).

## Results

Fifty four obese women (55.1%) had OSA that was mild in 70.4%, moderate in 25.9% and severe in 3.7%. Table 1 summarizes the clinical characteristics of PM and M obese women participating in the study. Compared to PM women, M women had higher levels of NHR and WHR (NHR: 0.24±0.02 vs 0.23±0.02; WHR 0.73±0.06 vs 0.69±0.06, p<0.05 for both) and a higher prevalence of OSA (64% vs 41%, p<0.05). M women had a worse glucose homeostasis (higher fasting glucose, 2 h glucose and lower insulinogenic index) than PM women only in non OSA groups. In OSA group glucose tolerance was comparable between PM and M women.

**Table 1:** Clinical, anthropometric, metabolic variables and nocturnal respiratory registration data in PM and M obese women according to the presence of OSA.

	Premenopausal women (n = 39)		Menopausal women (n = 59)	
	non OSA (n = 23)	*OSA (n = 16)*	non OSA (n = 21)	*OSA (n = 38)*
Age, yrs	37.5±9.5	*41.7±8.0*	58.5±8.4	*62.9±6.1* a
FH of diabetes, %	59.0	*25.0*	61.9	*36.8*
BMI, kg/m2	36.0±3.4§	*39.5±4.8* a	33.5±3.3	*36.8±3.7* b
Current smokers,%	13.6	*6.3*	23.8	*10.8*
Waist/height	0.68±0.05	*0.72±0.06* a	0.71±0.05	*0.74±0.06* a
Neck/height	0.23±0.02	*0.23±0.02*	0.23±0.02	*0.24±0.01*
Fat mass/fat free mass	0.9±0.2	*1.0±0.2*	0.9±0.2	*1.0±0.2*
Systolic BP, mmHg	128.2±20.3	*125.6±8.9*	129.3±11.8	*131.2±12.2*
Diastolic BP, mmHg	81.0±10.5	*80.6±4.4*	78.1±7.3	*80.8±7.0*
Heart rate, b/min	77.1±8.3	*81.1±13.5*	78.6±9.4	*74.4±9.6*
Fasting glucose, mg/dl	84.9±6.9§	*96.1±9.1* c	95.0±12.3	*93.6±9.6*
2 h glucose, mg/dl	98.8±24.3§	*116.1±22.2* a	122.0±43.4	*115.1±35.0*
ISI, mg/dl	9.2.3±6.4	*6.1±3.7* a	6.6±4.5	*6.7±4.7*
ΔI_30_/ΔG_30_,pmol/mmol	282.0±204.9§	*199.9±90.5*	111.2±76.3	*147.1±110.8*
CRP, mg/dl ^∧^	0.4 (0.2–0.7)	*0.9 (0.2–2.2)* #	0.4 (0.1–0.5)	*0.5 (0.3–0.7)* b
IFG/IGT, %	0/8.7	*18.8* b */12.5*	4.7/23.8	*21.1/13.2*
Hypertension, %	21.7	*31.3* #	47.6	*78.9* a
ESS score	5.7±4.0	*8.3±5.3*	6.9±4.8	*4.6±3.0*
AHI	0.8±1.1§§	*16.1±10.6* a	1.9±1.3	*14.4±7.9* c
ODI	1.2±1.2§§	*14.7±10.1* c	2.6±1.4	*14.2±8.5* c
Mean SaO_2_,%	96.1±1.3§§	*93.9±1.7* c	94.5±1.2	*93.7±1.4* a
Minimal SaO_2_,%	90.0±3.2§	*82.2±7.6* c	88.0±2.1	*80.9±4.7* c

FH: family history; BMI: body mass index; BP: blood pressure; IFG: impaired fasting glucose; IGT: impaired glucose tolerance; ISI: insulin sensitivity index; ΔI_30_/ΔG_30:_ insulinogenic index; CRP: C- reactive protein; ESS: Epworth Sleepiness Scale; AHI: Apnea-Hypopnea Index; ODI: Oxygen Desaturation Index; SaO_2_: nocturnal oxygen saturation. Data are expressed as mean ± SD except for CRP which is expressed as median (interquartile range).

a p<0.05,

b p<0.01,

c p<0.0001 OSA vs no OSA women;

§ p<0.05,

§§ p<0.001 no OSA premenopausal vs no OSA menopausal women;

# p<0.01 premenopausal OSA vs menopausal OSA women.

We then analyzed the differences between OSA and non OSA women separately in PM and M group. Among PM women, those with OSA had lower ISI and higher fasting glucose, 2 h glucose and IFG prevalence than non OSA women even after adjustment for BMI and WHR. Among M women, those with OSA were more frequently hypertensive and had higher CRP levels and similar glucose tolerance than non OSA women. (Table 1). The adjustment for BMI nullified the difference in CRP levels.

The proportion of hypersonnolence was not significantly higher in OSA than in non OSA women (in PM women 33 vs 14% and in M women 0% vs 20%, NS).

### Relationships between Nocturnal Polysomnographic Data and Clinical and Biochemical Variables

Table 2 shows the univariate correlations in the whole group of obese women. AHI, ODI and nocturnal SaO_2_ were significantly correlated with age and with all obesity indexes. Nocturnal mean and min SaO2 were associated with NHR, independently of the other obesity indexes (BMI, WHR and fat mass/fat-free mass) and age.

**Table 2:** Correlation coefficients of nocturnal respiratory registration data with obesity indexes, insulin sensitivity and fasting glucose in 98 obese women.

			Age	BMI	WHR	NHR	FM/FFM	FPG	2 hPG	ISI
AHI	r	.283^**^		.259^*^	.211	.258^*^	.228^*^	.067	.032	−.119
	p	.008		.015	.052	.017	.033	.533	.768	.292
ODI	r	.274^**^		.281^**^	.217^*^	.244^*^	.192	.111	.093	−.129
	p	.006		.005	.034	.018	.058	.276	.361	.224
Mean SaO2	r	−.456^**^		−.184	−.337^**^	−.349^**^	−.113	−.306^**^	−.183	.282^**^
	p	.000		.070	.001	.001	.268	.002	.072	.007
Min SaO2	r	−.359^**^		−.244^*^	−.188	−.263^*^	−.155	−.200^*^	−.155	.226^*^
	p	.000		.015	.068	.010	.128	.048	.127	.032
BMI	r	−.197		1	.529^**^	.403^**^	.504^**^	.059	.108	−.267^*^
	p	.051			.000	.000	.000	.563	.288	.011
WHR	r	.306^**^		.529^**^	1	.514^**^	.358^**^	.119	.122	−.260^*^
	p	.003		.000		.000	.000	.249	.239	.015
NHR	r	.311^**^		.403^**^	.514^**^	1	.098	.283^**^	.219^*^	−.404^**^
	p	.002		.000	.000		.347	.006	.034	.000
FM/FFM	r	−.021		.504^**^	.358^**^	.098	1	.003	.046	.003
	p	.837		.000	.000	.347		.979	.653	.977
FPG	r	.327^**^		.059	.119	.283^**^	.003	1	.585^**^	−.466^**^
	p	.001		.563	.249	.006	.979		.000	.000
2 hPG	r	.214^*^		.108	.122	.219^*^	.046	.585^**^	1	−.455^**^
	p	.035		.288	.239	.034	.653	.000		.000
ISI	r	−.148		−.267^*^	−.260^*^	−.404^**^	.003	−.466^**^	−.455^**^	1
	p	.165		.011	.015	.000	.977	.000	.000	

BMI: body mass index; WHR: waist/height ratio; NHR: neck/height ratio; FM/FFM: fat mass/fat free mass ratio; FPG: fasting glucose; 2 h PG: 2 h glucose; ISI: insulin sensitivity index; AHI: Apnea-Hypopnea Index; ODI: Oxygen Desaturation Index; mean SaO_2_: mean nocturnal oxygen saturation; min SaO_2_: minimal nocturnal oxygen saturation.

Both nocturnal mean and minimal SaO_2_ and NHR were correlated with ISI and fasting glucose.

In the multivariate analysis with ISI as dependent variable and BMI, WHR, NHR, mean and minimal nocturnal SaO_2_ as independent variables, only NHR remained independently associated with ISI (β −0.404, p<0.0001). In a model with fasting glucose as dependent variable and age, WHR, NHR, mean and minimal nocturnal SaO2 as independent variables, only mean nocturnal SaO_2_ remained independently associated with fasting glucose (β −0.310, p<0.0001).

No relations were found between nocturnal polisomnographic data and ESS score, CRP, BP and insulinogenic index. Systolic BP was correlated with age (0.225, p<0.05), NHR, WHR and ISI (r 0.246, r 0.236 and r −0.212, p<0.05 for all), however after controlling for age these relations became not significant.

### Effects of Lifestyle Intervention in 54 Obese Women with OSA

**Figure 1:**
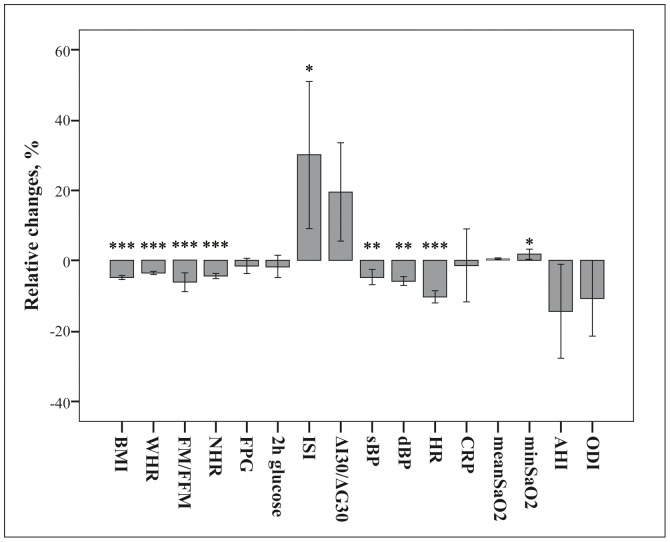
Relative changes in anthropometric, metabolic and nocturnal registration respiratory data in 54 non diabetic obese women with OSA after lifestyle intervention. BMI: body mass index; WHR: waist/height ratio; FM/FFM: fat mass/fat free mass ratio; NHR: neck/height ratio; FPG: fasting glucose; ISI: insulin sensitivity index; ΔI_30_/ΔG_30:_ insulinogenic index; sBP: systolic blood pressure; dBP: diastolic blood pressure; HR: heart rate; CRP: C-reactive protein; min SaO_2_: minimal nocturnal oxygen saturation; AHI: Apnea-Hypopnea Index; ODI: Oxygen Desaturation Index. Data are expressed as mean±ES. ***p<0.0001, **p<0.001, *p<0.05.

After 3 months of lifestyle intervention, obese women with OSA (16 PM and 38 M) reduced their weight by 4.8±2.7% (p<0.0001). This was associated with significant reduction of WHR, NHR and BP, improvement in body composition, insulin sensitivity and β cell function and increase in minimal nocturnal SaO_2_ (Figure 1). Remission of OSA (AHI <5) was observed in 23% of women. AHI decreased by 17% and ODI by 14%, but these changes were not statistically significant probably because of a high individual variability.

The effect of lifestyle intervention on polysomnographic data was independent of age and baseline values of nocturnal respiratory data, obesity indexes and cardio metabolic variables.

Changes in minimal nocturnal SaO_2_ were correlated with those in weight and fat mass/fat free mass (r −0.273 and r −0.367, p<0.05). Changes in ISI and fasting glucose were associated with those in minimal nocturnal SaO_2_ (r 0.400, r −0.324, p<0.05 for both) and not with those of all obesity indexes.

## Discussion

Our study provides evidence that 1) in non diabetic obese women, OSA is frequent also before menopausal status where it is associated with alterations of glucose homeostasis, 2) lower nocturnal mean SaO_2_ and higher neck circumference have additive effects in the worsening of glucose tolerance, 3) a modest weight reduction is able to normalize the nocturnal breathing pattern in approximately one quarter of OSA women, 4) in OSA women, the lifestyle intervention-induced increase in minimal nocturnal SaO_2_ is associated with the improvement of glucose tolerance_._

The frequency of OSA we found in PM women was comparable to the 40% observed in severely obese PM women in a Norwegian obesity center [10]. This prevalence was about four fold higher than that estimated in US obese women of similar age using the same definition of OSA (11%) [28] likely because general population was the setting of recruitment in the U.S. study.

The novel result of the present study is that glucose homeostasis worsens in the presence of OSA more clearly in PM than in M women. This finding is in agreement with the previous demonstration that OSA prevalence significantly increases with the worsening of glucose tolerance in PM but not in M women [8]. The association of menopause with worse glucose tolerance in non OSA group together with the comparable glucose tolerance in OSA and non OSA.

M women, suggests that the negative effect on glucose tolerance exerted by increasing age [29] might have masked a possible additive effect of OSA on glucose homeostasis.

The results of our study support the deleterious effects of nocturnal hypoxia on glucose tolerance. Acute hypoxemia and prolonged exposure to high altitude hypobaric hypoxia alter glucose metabolism in healthy subjects [30], [31]. Further, glucose tolerance is impaired in patients with chronic obstructive pulmonary disease and improves with oxygen supplementation [32].

Relatively few studies investigated the relationships between nocturnal respiratory data and metabolic variables. Insulin and glucose levels were found associated with AHI and/or ODI and/or nocturnal SaO_2_ but this was often dependent on obesity [13], [33]–[35]. In the only study available in women, low nocturnal minimal SaO_2_ was associated with decreased insulin sensitivity and higher glucose levels independently on waist/hip ratio in overweight women aged 20–70 years [11].

Hypoxemia is an important stimulus for increasing sympathetic activity which in turn can impair glucose homeostasis by increasing glycogen breakdown and gluconeogenesis and for releasing inflammatory factors with deleterious effects on insulin synthesis and peripheral action [36]. The independent relation we found between meanSaO2 and fasting glucose suggests that nocturnal hypoxia may increase glucose levels not only by worsening insulin sensitivity but also exerting a direct hyperglycemic effect. In support of this hypothesis, it had been demonstrated that in humans, a physiological increase in epinephrine concentration induces a sustained and biphasic increase in glucose production independent of changes in plasma insulin concentrations [37].

The reason why nocturnal hypoxia severity is more important than the frequency of nocturnal desaturations in determining glucose homeostasis alterations has yet to be defined.

We observed a negative and independent relation of NHR with insulin sensitivity in accord with what reported in the Framingham Heart Study [38]. This finding suggests that an increased neck circumference may contribute to impair glucose tolerance. The mechanism by which a greater neck circumference could worsen insulin sensitivity may be the mechanical compression of the upper airway with consequent induction of nocturnal hypoxia and/or the overproduction of free fatty acids. Neck circumference indeed is a surrogate measure of upper-body subcutaneous adipose tissue which is responsible for a much larger proportion of systemic free fatty acid release than visceral fat, particularly in obese individuals [39] and in women who store larger proportions of free fatty acids than men in subcutaneous adipose tissue [38], [40].

We demonstrated that a lifestyle intervention is able to normalize in a short time the nocturnal respiratory pattern in nearly a quarter of obese women with mild OSA. We observed a relation between changes in obesity and in minimal nocturnal SaO_2_ but not in AHI. This result agrees with those of most dietary intervention studies and is likely due to the large individual variability in AHI changes [22]. Notably, AHI decreased of the same magnitude (14%) reported in subjects who experienced a 5% weight loss in a 4-years observational study [4]. As observed in previous studies, AHI changes were unrelated to those of metabolic variables [20], [41] whereas changes in minimal SaO2 were associated with changes in glycaemia and insulin sensitivity. The demonstration that in the obese OSA women investigated in this study, the improvement in glucose tolerance was not related to the reduction of obesity, supports the hypothesis that the decrease of nocturnal hypoxia is the mediator of the weight loss induced improvement of glucose tolerance in OSA women.

In conclusion in obese women, glucose homeostasis worsens due to the additive effects of nocturnal hypoxia and increased neck circumference through mechanisms partially independent of obesity (sympathetic activation and mechanical narrowing of the upper airway). OSA is more clearly associated with glucose intolerance in premenopausal than in menopausal women. In OSA women, the improvement of nocturnal hypoxia induced by lifestyle modifications is associated with that of glucose homeostasis.

## References

[1] Young T, Palta M, Dempsey J, Peppard PE, Nieto FJ, et al (2009) Burden of sleep apnea: rationale, design, and major findings of the Wisconsin Sleep Cohort study. WMJ 108: 246–249. 19743755 PMC2858234

[2] Bradley TD, Floras JS (2009) Obstructive sleep apnoea and its cardiovascular consequences. Lancet 373: 82–93. 19101028 10.1016/S0140-6736(08)61622-0

[3] Pamidi S, Aronsohn RS, Tasali E (2010) Obstructive sleep apnea: role in the risk and severity of diabetes. Best Pract Res Clin Endocrinol Metab 24: 703–715. 21112020 10.1016/j.beem.2010.08.009 PMC2994098

[4] Peppard PE, Young T, Palta M, Dempsey J, Skatrud J (2000) Longitudinal study of moderate weight change and sleep-disordered breathing. JAMA 284: 3015–3021. 11122588 10.1001/jama.284.23.3015

[5] Shaw JE, Punjabi NM, Wilding JP, Alberti KG, Zimmet PZ, et al (2008) Sleep-disordered breathing and type 2 diabetes: a report from the International Diabetes Federation Taskforce on Epidemiology and Prevention. Diabetes Res Clin Pract 81: 2–12. 18544448 10.1016/j.diabres.2008.04.025

[6] Reichmuth KJ, Austin D, Skatrud JB, Young T (2005) Association of sleep apnea and type II diabetes: a population-based study. Am J Respir Crit Care Med 172: 1590–1595. 16192452 10.1164/rccm.200504-637OC PMC2718458

[7] Botros N, Concato J, Mohsenin V, Selim B, Doctor K, et al (2009) Obstructive sleep apnea as a risk factor for type 2 diabetes. Am J Med 122: 1122–1127. 19958890 10.1016/j.amjmed.2009.04.026 PMC2799991

[8] Foster GD, Borradaile KE, Sanders MH, Millman R, Zammit G, et al (2009) A randomized study on the effect of weight loss on obstructive sleep apnea among obese patients with type 2 diabetes: the Sleep AHEAD study Arch Intern Med. 169: 1619–1626. 10.1001/archinternmed.2009.266 PMC2879275 19786682

[9] Seicean S, Kirchner HL, Gottlieb DJ, Punjabi NM, Resnick H, et al (2008) Sleep-disordered breathing and impaired glucose metabolism in normal-weight and overweight/obese individuals: the Sleep Heart Health Study. Diabetes Care 31: 1001–1006. 18268072 10.2337/dc07-2003

[10] Fredheim JM, Rollheim J, Omland T, Hofsø D, Røislien J, et al (2011) Type 2 diabetes and pre-diabetes are associated with obstructive sleep apnea in extremely obese subjects: a cross-sectional study. Cardiovasc Diabetol 10: 84–89. 21943153 10.1186/1475-2840-10-84 PMC3206416

[11] Theorell-Haglöw J, Berne C, Janson C, Lindberg E (2008) Obstructive sleep apnoea is associated with decreased insulin sensitivity in females. Eur Respir J 31: 1054–1060. 18184681 10.1183/09031936.00074907

[12] Togeiro SM, Carneiro G, Ribeiro Filho FF, Zanella MT, Santos-Silva R, et al.. (2012) Consequences of Obstructive Sleep Apnea on Metabolic Profile: A Population-Based Survey. Obesity (Silver Spring) doi:10.1038/oby.2012.146. 10.1002/oby.20288 23712988

[13] Bulcun E, Ekici M, Ekici A (2012) Disorders of glucose metabolism and insulin resistance in patients with obstructive sleep apnoea syndrome. Int J Clin Pract. 66: 91–97. 10.1111/j.1742-1241.2011.02795.x 22171909

[14] Davies RJ, Turner R, Crosby J, Stradling JR (1994) Plasma insulin and lipid levels in untreated obstructive sleep apnoea and snoring; their comparison with matched controls and response to treatment. J Sleep Res. 3: 180–185. 10.1111/j.1365-2869.1994.tb00126.x 10607124

[15] Barceló A, Barbé F, Llompart E, Mayoralas LR, Ladaria A, et al (2004) Effects of obesity on C-reactive protein level and metabolic disturbances in male patients with obstructive sleep apnea. Am J Med. 117: 118–121. 10.1016/j.amjmed.2004.01.025 15234648

[16] Gruber A, Horwood F, Sithole J, Ali NJ, Idris I (2006) Obstructive sleep apnoea is independently associated with the metabolic syndrome but not insulin resistance state. Cardiovasc Diabetol. 5: 22–29. 10.1186/1475-2840-5-22 PMC1636630 17078884

[17] Sharma SK, Kumpawat S, Goel A, Banga A, Ramakrishnan L, et al (2007) Obesity, and not obstructive sleep apnea, is responsible for metabolic abnormalities in a cohort with sleep-disordered breathing. Sleep Med 8: 12–17. 17157064 10.1016/j.sleep.2006.06.014

[18] Blaak E (2008) Sex differences in the control of glucose homeostasis. Curr Opin Clin Nutr Metab care 11: 500–504. 18542013 10.1097/MCO.0b013e32830467d3

[19] Franzini L, Ardigò D, Cavalot F, Miccoli R, Rivellese AA, et al.. (2012) Women show worse control of type 2 diabetes and cardiovascular disease risk factors than men: Results from the MIND.IT Study Group of the Italian Society of Diabetology. Nutr Metab Cardiovasc Dis. doi:0.1016/j.numecd.2011.12.003. 10.1016/j.numecd.2011.12.003 22397873

[20] Tuomilehto HP, Seppä JM, Partinen MM, Peltonen M, Gylling H, et al (2009) Lifestyle intervention with weight reduction: first-line treatment in mild obstructive sleep apnea. Am J Respir Crit Care Med 179: 320–327. 19011153 10.1164/rccm.200805-669OC

[21] Haines KL, Nelson LG, Gonzalez R, Torrella T, Martin T, et al (2007) Objective evidence that bariatric surgery improves obesity-related obstructive sleep apnea. Surgery 141: 354–358. 17349847 10.1016/j.surg.2006.08.012

[22] Anandam A, Akinnusi M, Kufel T, Porhomayon J, El-Solh AA (2012) Effects of dietary weight loss on obstructive sleep apnea: a meta-analysis. Sleep Breath doi: 10.1007/s11325–012–0677–3. 10.1007/s11325-012-0677-3 22374151

[23] Bixler EO, Vgontzas AN, Lin HM, Ten Have T, Rein J, et al (2001) Prevalence of sleep-disordered breathing in women: effects of gender. Am J Respir Crit Care Med 163: 608–613. 11254512 10.1164/ajrccm.163.3.9911064

[24] Johns MW (1991) A new method for measuring daytime sleepiness: the Epworth sleepiness scale. Sleep 14: 540–545. 1798888 10.1093/sleep/14.6.540

[25] Matsuda M, DeFronzo RA (1999) Insulin sensitivity indices obtained from oral glucose tolerance testing: comparison with the euglycemic insulin clamp. Diabetes Care 22: 1462–1470. 10480510 10.2337/diacare.22.9.1462

[26] Phillips DI, Clark PM, Hales CN, Osmond C (1194) Understanding oral glucose tolerance: comparison of glucose or insulin measurements during the oral glucose tolerance test with specific measurements of insulin resistance and insulin secretion. Diabet Med. 11: 286–292. 10.1111/j.1464-5491.1994.tb00273.x 8033528

[27] Iber C, Ancoli-Israel S, Chesson AL, Quan SF (2007) The AASM manual for the scoring of sleep and associated events: rules, terminology, and technical specifications. 1st ed. Westchester, IL: American Academy of Sleep Medicine. 59 p.

[28] Young T, Peppard PE, Taheri S (2005) Excess weight and sleep-disordered breathing. J Appl Physiol 99: 1592–1599. 16160020 10.1152/japplphysiol.00587.2005

[29] De Fronzo RA (2009) Banting Lecture. From the triumvirate to the ominous octet: a new paradigm for the treatment of type 2 diabetes mellitus. Diabetes 58: 773–795. 19336687 10.2337/db09-9028 PMC2661582

[30] Oltmanns KM, Gehring H, Rudolf S, Schultes B, Rook S, et al (2004) Hypoxia causes glucose intolerance in humans. Am J Respir Crit Care Med 169: 1231–1237. 15044204 10.1164/rccm.200308-1200OC

[31] Larsen JJ, Hansen JM, Olsen NV, Galbo H, Dela F (1997) The effect of altitude hypoxia on glucose homeostasis in men. J Physiol 504: 241–249. 9350634 10.1111/j.1469-7793.1997.241bf.x PMC1159952

[32] Jakobsson P, Jorfeldt L (2006) Oxygen supplementation increases glucose tolerance during euglycaemic hyperinsulinaemic glucose clamp procedure in patients with severe COPD and chronic hypoxaemia. Clin Physiol Funct Imaging 26: 271–274. 16939503 10.1111/j.1475-097X.2006.00686.x

[33] Elmasry A, Lindberg E, Berne C, Janson C, Gislason T, et al (2001) Sleep-disordered breathing and glucose metabolism in hypertensive men: a population-based study. J Intern Med 249: 153–161. 11240844 10.1046/j.1365-2796.2001.00787.x

[34] Ip MS, Lam B, Ng MM, Lam WK, Tsang KW, et al (2002) Obstructive sleep apnea is independently associated with insulin resistance. Am J Respir Crit Care Med 165: 670–676. 11874812 10.1164/ajrccm.165.5.2103001

[35] Kapsimalis F, Varouchakis G, Manousaki A, Daskas S, Nikita D, et al (2008) Association of sleep apnea severity and obesity with insulin resistance, C-reactive protein, and leptin levels in male patients with obstructive sleep apnea. Lung 186: 209–217. 18365276 10.1007/s00408-008-9082-x

[36] Punjabi NM, Polotsky VY (2005) Disorders of glucose metabolism in sleep apnea. J Appl Physiol 99: 1998–2007. 16227461 10.1152/japplphysiol.00695.2005

[37] Dufour S, Lebon V, Shulman G I, Petersen KF (2009) Regulation of net hepatic glycogenolysis and gluconeogenesis by epinephrine in humans. Am J Physiol Endocrinol Metab 297: E231–E235. 19458062 10.1152/ajpendo.00222.2009 PMC2711660

[38] Preis SR, Massaro JM, Hoffmann U, D’Agostino RB Sr, Levy D, et al (2010) Neck circumference as a novel measure of cardiometabolic risk: the Framingham Heart study. J Clin Endocrinol Metab 95: 3701–3710. 20484490 10.1210/jc.2009-1779 PMC2913042

[39] Nielsen S, Guo Z, Johnson CM, Hensrud DD, Jensen MD (2004) Splanchnic lipolysis in human obesity. J Clin Invest 113: 1582–1588. 15173884 10.1172/JCI21047 PMC419492

[40] Koutsari C, Snozek CL, Jensen MD (2008) Plasma NEFA storage in adipose tissue in the postprandial state: sex-related and regional differences. Diabetologia 51: 2041–2048. 18712345 10.1007/s00125-008-1126-5 PMC2637373

[41] Nerfeldt P, Nilsson BY, Mayor L, Uddén J, Friberg D (2010) A two-year weight reduction program in obese sleep apnea patients. J Clin Sleep Med 6: 479–486. 20957850 PMC2952753

